# Do Treatment Quality Indicators Predict Cardiovascular Outcomes in Patients with Diabetes?

**DOI:** 10.1371/journal.pone.0078821

**Published:** 2013-10-30

**Authors:** Grigory Sidorenkov, Jaco Voorham, Dick de Zeeuw, Flora M. Haaijer-Ruskamp, Petra Denig

**Affiliations:** 1 Department of Clinical Pharmacology, University of Groningen, University Medical Center Groningen, Groningen, The Netherlands; 2 Research Institute SHARE of the Graduate School of Medical Sciences, University of Groningen, University Medical Center Groningen, Groningen, The Netherlands; University of Leicester, United Kingdom

## Abstract

**Background:**

Landmark clinical trials have led to optimal treatment recommendations for patients with diabetes. Whether optimal treatment is actually delivered in practice is even more important than the efficacy of the drugs tested in trials. To this end, treatment quality indicators have been developed and tested against intermediate outcomes. No studies have tested whether these treatment quality indicators also predict hard patient outcomes.

**Methods:**

A cohort study was conducted using data collected from >10.000 diabetes patients in the Groningen Initiative to Analyze Type 2 Treatment (GIANTT) database and Dutch Hospital Data register. Included quality indicators measured glucose-, lipid-, blood pressure- and albuminuria-lowering treatment status and treatment intensification. Hard patient outcome was the composite of cardiovascular events and all-cause death. Associations were tested using Cox regression adjusting for confounding, reporting hazard ratios (HR) with 95% confidence intervals.

**Results:**

Lipid and albuminuria treatment status, but not blood pressure lowering treatment status, were associated with the composite outcome (HR = 0.77, 0.67–0.88; HR = 0.75, 0.59–0.94). Glucose lowering treatment status was associated with the composite outcome only in patients with an elevated HbA1c level (HR = 0.72, 0.56–0.93). Treatment intensification with glucose-lowering but not with lipid-, blood pressure- and albuminuria-lowering drugs was associated with the outcome (HR = 0.73, 0.60–0.89).

**Conclusion:**

Treatment quality indicators measuring lipid- and albuminuria-lowering treatment status are valid quality measures, since they predict a lower risk of cardiovascular events and mortality in patients with diabetes. The quality indicators for glucose-lowering treatment should only be used for restricted populations with elevated HbA1c levels. Intriguingly, the tested indicators for blood pressure-lowering treatment did not predict patient outcomes. These results question whether all treatment indicators are valid measures to judge quality of health care and its economics.

## Introduction

Patients with type 2 diabetes are at high risk for cardiovascular morbidity and mortality, and often require treatment with drugs. Treatment is aimed at reducing risk factors, such as high glucose, blood pressure and lipid levels, with the ultimate goal to reduce morbidity and mortality. A novel drug therapy showing a 15–25% relative risk reduction in hard outcomes is considered to be a large success [1]–[4]. Such evidence-based therapies are usually integrated in guidelines which define optimal treatment. However, guideline implementation is difficult, and 10 to 55% of patients with diabetes and elevated risk factors levels are not adequately treated [5],[6]. Improvement of treatment in clinical practice thus has the potential of a large reduction in morbidity and mortality. The quality of treatment is as important as the drugs being prescribed, but there is lack of knowledge on how best to measure treatment quality. Therefore, valid treatment quality indicators are needed that can be implemented in clinical practice and reflect treatment effects.

Several treatment quality indicators for cardiovascular risk management have been proposed by quality improvement organizations [7]–[9]. They measure the percentage of patients with a certain treatment status, that is, patients receiving or not receiving a specific medication at one point in time. As alternative, clinical action indicators have been proposed [10]–[12], which measure the percentage of patients in whom treatment is started or intensified when indicated. Before implementation, it is important to know whether treatment as measured by means of the quality indicators is predictive of better patient outcomes. Although there is an extensive evidence from clinical trials that better treatment leads to better outcomes [1]–[4], poorly defined treatment quality indicators or indicators using wrong assumptions are not likely to result in better patient outcomes. Such indicators may inadequately capture the indication for treatment, or be too simplistic to reflect treatment quality over time.

Previous studies tested whether treatment quality indicators are predictive of better intermediate outcomes in patients with diabetes. It was found that indicators measuring glucose or cholesterol lowering treatment status showed predictive value on intermediate outcomes, that is, better glycemic and cholesterol control [5],[6],[12],[13]. The indicators measuring whether treatment was started or intensified in uncontrolled patients, showed predictive value for glycemic, as well as blood pressure and cholesterol control. Although these intermediate outcomes are considered to be predictors of cardiovascular events [14]–[17], the direct relationship between treatment quality indicators and hard outcomes is unknown [18].

The aim of this study is to test which treatment quality indicators are predictive of a lower risk of cardiovascular outcomes in patients with type 2 diabetes. We conducted a cohort study measuring the treatment quality in primary care using various indicators and assessing their relation to patient outcomes in a follow-up period of three years. The indicators predictive of better hard outcomes were identified.

## Methods

Patients who had been diagnosed with type 2 diabetes before 1 January 2007 were selected from the GIANTT (Groningen Initiative to Analyze Type 2 Diabetes) database [19]. This regional longitudinal database contains anonymized data extracted from electronic medical records (EMR) of type 2 diabetes patients from the north of the Netherlands who are managed in primary care. These records include prescription data, medical history, routine laboratory test results and physical examinations. Medical history data includes diagnoses, which are documented in the medical records by means of the International Classification of Primary Care (ICPC) [20] or short text descriptions which were manually coded in GIANTT.

Data on patient cardiovascular events and mortality were collected from the Dutch Hospital Data register and municipality register provided by the Central Bureau of Statistics in the Netherlands [21]. These data include patients discharge diagnoses that are coded according to the International Classification of Diseases-9-Clinical Modification, hospital procedure codes that are coded according to the Classification of Medical Procedures developed by the Central Administration of Procedures in the Netherlands, and mortality information.

### Quality indicators

We selected a range of commonly used and recommended quality indicators for treatment of cardiovascular risk factors in patients with diabetes from national indicator sets and a previous review study [7]–[10]. The complete list of the fourteen included quality indicators and their definitions is presented in Table 1. The treatment quality was measured in the year 2007.

**Table 1:** Definitions of quality indicators.

Treatment quality indicators	Baseline factor	Definition of quality
**HbA1c**		
Diabetes patients with HbA1c test who are treated with glucose lowering drug(s)	First HbA1c test in 2007	Glucose lowering drug prescription within last 3 months of 2007
Diabetes patients with HbA1c >7% who are treated with glucose lowering drug(s)	First HbA1c test in 2007 if value >7%	Glucose lowering drug prescription within last 3 months of 2007
Diabetes patients with HbA1c >7% not on maximum treatment receiving glucose lowering treatment intensification	First HbA1C test in 2007 if value >7%	Glucose lowering drug start or dosage increase within 180 days after baseline test
Diabetes patients with HbA1c >8.5% not on maximum treatment receiving glucose lowering treatment intensification	First HbA1c test in 2007 if value >8.5%	Glucose lowering drug start or dosage increase within 180 days after baseline test
**Low-density lipoprotein cholesterol (LDL-C)**		
Diabetes patients with LDL-C test who are treated with lipid lowering drugs	First LDL-C test in 2007	Lipid lowering drug prescription within last 3 months of 2007
Diabetes patients with LDL-C >2.5 mmol/l not on maximum treatment receiving lipid lowering treatment intensification	First LDL-C test in 2007 if value >2.5 mmol/l	Lipid lowering drug start or dosage increase within 180 days after baseline test
Diabetes patients with LDL-C >3.5 mmol/l not on maximum treatment receiving lipid lowering treatment intensification	First LDL-C test in 2007 if value >3.5 mmol/l	Lipid lowering drug start or dosage increase within 180 days after baseline test
**Systolic blood pressure (SBP)**		
Diabetes patients with SBP ≥140 mm Hg who are treated with blood pressure lowering drug(s)	First SBP test in 2007 if value ≥140 mm Hg	Blood pressure lowering drug prescription within last 3 months of 2007
Diabetes patients with SBP ≥140 mm Hg not on maximum treatment receiving blood pressure lowering treatment intensification	First SBP test in 2007 if value ≥140 mm Hg	Blood pressure lowering drug start or dosage increase within 180 days after baseline test
Diabetes patients with SBP ≥160 mm Hg not on maximum treatment receiving blood pressure lowering treatment intensification	First SBP test in 2007 if value ≥160 mm Hg	Blood pressure lowering drug start or dosage increase within 180 days after baseline test
Diabetes patients with 2 sequential SBP ≥140 mm Hg receiving blood pressure lowering treatment intensification	First SBP test in 2007 with value ≥140 mm Hg	Blood pressure lowering drug start or dose increase within 180 days after baseline test
Diabetes patients with 2 sequential SBP ≥160 mm Hg receiving blood pressure lowering treatment intensification	First SBP test in 2007 with value ≥160 mm Hg	Blood pressure lowering drug start or dose increase within 180 days after baseline test
**Albumin:creatinine ratio (ACR)**		
Diabetes patients with ≥2.5 mg/mmol (males) or ≥3.5 mg/mmol (females) treated with ACE-inhibitors or angiotensin receptor blocker (ARB)	First ACR test in 2007 if value ≥2.5 mg/mmol (males) or ≥3.5 mg/mmol (females)	ACE-i or ARB drug prescription within last 3 months of 2007
Patients with ACR ≥2.5 mg/mmol (males) or ≥3.5 mg/mmol (females) receiving ACE-inhibitors or ARB treatment intensification	First ACR test in 2007 if value ≥2.5 mg/mmol (males) or ≥3.5 mg/mmol (females)	ACE-i or ARB start or dosage increase within 180 days after baseline test

The indicators of current treatment status measured whether (1) patients with diabetes are treated with glucose- or lipid-lowering drugs, (2) patients with diabetes and elevated levels of HbA1c, systolic blood pressure (SBP) or albumin:creatinine ratio (ACR) are treated with glucose-, blood pressure- or albuminuria-lowering drugs. A patient was considered as being treated when a prescription was recorded within the last three months of the measurement year, since a single prescription can be issued for a maximum period of 3 months in The Netherlands.

The indicators of treatment intensification measured whether patients with diabetes and an elevated risk factor level received a start or intensification of pharmacotherapy. According to the Dutch guidelines in 2007, such treatment intensification was recommended for patients with levels of HbA1c>7% (53 mmol/mol); low density lipoprotein cholesterol (LDL-C)>2.5 mmol/l; SBP> = 140 mm Hg; and ACR> = 2.5 mg/mmol (males) and> = 3.5 mg/mmol (females) [22]. Since treatment quality might be more at stake at higher thresholds, we also included treatment indicators focusing on patients with more elevated risk factor levels of HbA1c>8.5% (69 mmol/mol), LDL-C>3.5 mmol/l, and SBP> = 160 mmHg [23],[24]. Moreover, since doctors may wait for a confirmation blood pressure reading before intensifying treatment, we included the indicators measuring treatment intensification after two sequentially elevated blood pressure levels within 150 days [10].

A patient was considered as receiving treatment intensification when a new drug class was started or added, or a dosage was increased within a period of 180 days after the first elevated risk factor level in 2007. Switches between drugs were not included as intensification. The included drug classes are presented in Table S1.

Patients on maximum treatment were excluded from the intensification indicators, since there is no room for further intensification of drug treatment in primary care setting. We defined maximum treatment according to the Dutch guideline for primary care. The following treatment was considered as maximum treatment: for glucose lowering treatment, the use of insulin; for lipid lowering treatment, the use of one or more drugs at maximum maintenance dosage; for blood pressure lowering treatment, the use of 3 or more drugs classes at maximum maintenance dosage; for albuminuria lowering treatment, prescribing of either angiotensin-converting enzyme inhibitor (ACE-i) or an angiotensin-II-receptor blockers (ARB) at maximum dosage. Dosage recommendations were obtained from the Dutch Pharmacotherapy Compendium [25].

### Outcomes

The outcome of this study was a composite of cardiovascular events, including myocardial infarction, stroke, transient ischemic attack, coronary revascularization procedures, peripheral vascular complications, and all-cause death. The complete list of included events is presented in Table S2.

### Statistical analysis

Using standardized differences, we compared baseline characteristics of patients receiving with those not receiving treatment according to a quality indicator. Follow-up time and diabetes duration are presented as median with interquartile ranges (IQR). Diabetes duration is categorized to <3, 3–10, and >10 years for further analyses, since recently diagnosed patients are assumed to be different from those having diabetes for many years with regard to treatment decision. This difference is not expected to be proportional to diabetes duration. Other continuous variables are presented as means with standard deviations (SD). We used Cox Proportional Hazards regression to test the association between each of the included quality indicators and the outcomes. The provision of treatment or treatment intensification according to the quality indicator was defined as binary independent variable at patient level. Outcome risk was measured from the index date to the event date. For patients receiving treatment according to the quality indicator, the index date was the date of the last prescription or the date of treatment intensification in 2007. For the control group, the index date was a randomly generated date computed according to the observed distribution of treatment prescription dates of patients with treatment or treatment intensification in 2007. Patients who were lost due to changes in place of residence in the year of quality measurement (2007) were excluded from the analysis. Patients who were lost due to changes in place of residence during the follow-up period (2008–2010) were censored. Patients with missing baseline risk factor test in 2007 were excluded from the analyses per indicator, since treatment and treatment intensification were computed in relation to a risk factor test.

We tested the proportional hazards assumption for each covariate in all models by examining scatterplots of residuals against hazard time, which revealed no violation of the proportionality assumption.

### Confounding

For each indicator, a crude model adjusting only for baseline history of cardiovascular events, and a fully adjusted model including other patient characteristics were built. Most importantly, this adjustment is needed to reduce confounding by indication, that is, patients who are sicker are likely to be treated more aggressively but may still have worse outcomes, leading to negative associations. These patient characteristics are age, gender, diabetes duration, baseline risk factor level, baseline treatment status (glucose-, blood pressure-, lipid-lowering drugs), history of malignancies, and history of psychological disorders. The complete list of baseline cardiovascular morbidity and concomitant diseases is presented in Table S3.

### Ethics statement

In The Netherlands, according to the Code of Conduct for the use of data in Health Research (“Gedragscode gezondheidsonderzoek” approved in 2004 by the Dutch College for Protection of Personal Data, taking into account Article 25 of the Dutch Act on the Protection of Personal Data) no ethics committee approval was required for this research using data from anonymous medical records.

## Results

A cohort of 10058 patients with type 2 diabetes was eligible for this study, excluding 893 patients who were not identifiable in the Dutch Hospital Data register and 74 patients for whom disease duration was missing. Baseline patient characteristics are shown in Table 2. Depending on the eligibility criteria, the number of patients included per indicator ranged from 401 to 8455 (Table 3). The median follow-up ranged from 3.1 to 3.6 years across the models, which tested the associations between the each of the fourteen quality indicators and hard outcomes (mean of the medians follow-up 3.3 years (IQR 3.1–3.5). Percentages of patients who had a cardiovascular outcome during follow-up varied from 15.2% to 30.6% across the models. Baseline patients characteristics per indicator are presented in Table S4. In general, patients who received treatment for a specific risk factor were older, with a longer diabetes duration, had more related comorbidity and comedication. Patients with elevated risk factor levels receiving treatment intensification, in turn, were generally younger, with a shorter diabetes duration, less related comorbidity and comedication, and had a higher baseline risk factor level. The numbers of patients receiving treatment according to quality indicators and the number of events per indicator are presented in Table 3. Generally, treatment levels were high, whereas treatment intensification levels were low (Table 3).

**Table 2:** Patient characteristics at baseline (n = 10058).

Patient characteristics	Number of patients with observation (%)	Mean ± standard deviation
Age (years)		66.7±12.2
Male gender	4805 (47.8)	
Diabetes duration (years)		4 (2; 8)*
<3	3466 (34.5)	
3-10	5091 (50.6)	
>10	1501 (14.9)	
HbA1c (%(mmol/mol))	8602 (85.6)	6.9 (52)±1.0 (8)
LDL-cholesterol (mmol/l)	6587 (65.5)	2.4±0.9
Systolic blood pressure (mm Hg)	8596 (85.5)	143.1±20.8
Albumin:creatinine ratio (mg/mmol)	4699 (46.7)	5.0±15.1
Treated with glucose lowering drugs	8450 (84.0)	
Treated with lipid lowering drugs	7466 (74.2)	
Treated with blood pressure lowering drugs	7587 (75.4)	
History of cardiovascular morbidity	1970 (19.6)	
History of malignancy	721 (7.2)	
History of psychological comorbidity	379 (3.8)	

* - median (25^th^ and 75^th^ percentiles).

**Table 3:** Predictive value of quality indicators on a composite of cardiovascular events and all-cause death represents the hazard of event occurrence in patients treated as defined by the quality indicator in comparison to those not treated as such.

Quality indicators	Treated according to QI (yes/no)	Number/% of patient treated according to QI	Number of patients with events	Hazard ratio in crude model*	Hazard ratio in adjusted model**
Treated with glucose lowering drugs	Yes	6754 (79,9%)	1225	0.93 (0.82; 1.05)	0.91 (0.80; 1.03)
	No	1701 (20,1%)	320		
Treated with glucose lowering drugs in patients with HbA1c >7 (%)	Yes	2462 (91,2%)	497	**0.66 (0.51; 0.85)**	**0.72 (0.56; 0.93)**
	No	238 (8,8%)	70		
Treatment intensification in patients with HbA1c >7 (%)	Yes	848 (34,5%)	135	**0.65 (0.53; 0.79)**	**0.73 (0.60; 0.89)**
	No	1607 (65,5%)	386		
Treatment intensification in patients with HbA1c >8.5 (%)	Yes	145 (36,2%)	26	0.80 (0.50; 1.28)	0.75 (0.47; 1.23)
	No	256 (63,8%)	56		
Treated with lipid lowering drugs	Yes	4360 (67,4%)	662	**0.65 (0.58; 0.73)**	**0.77 (0.67; 0.88)**
	No	2111 (32,6%)	442		
Treatment intensification in patients with LDL-C >2.5 (mmol/l)	Yes	375 (16,7%)	59	0.84 (0.64; 1.11)	1.06 (0.80; 1.42)
	No	1864 (83,3%)	343		
Treatment intensification in patients with LDL-C >3.5 (mmol/l)	Yes	184 (26,9%)	36	1.13 (0.76; 1.66)	1.43 (0.96; 2.13)
	No	499 (73,1%)	87		
Treated with blood pressure lowering drugs in patients with SBP ≥140 (mmHg)	Yes	3915 (79,3%)	803	1.12 (0.95; 1.32)	1.07 (0.91; 1.27)
	No	1022 (20,7%)	172		
Treatment intensification in patients with SBP ≥140 (mmHg)	Yes	1004 (20,6%)	216	1.05 (0.90; 1.22)	1.02 (0.88; 1.20)
	No	3860 (79,4%)	795		
Treatment intensification in patients with 2 sequential SBP tests ≥140 (mmHg)	Yes	982 (23,7%)	210	1.05 (0.90; 1.23)	1.07 (0.91; 1.26)
	No	3164 (76,3%)	647		
Treatment intensification in patients with SBP ≥160 (mmHg)	Yes	598 (30,7%)	140	0.98 (0.81; 1.20)	1.00 (0.82; 1.23)
	No	1349 (69,3%)	324		
Treatment intensification in patients with 2 sequential SBP tests ≥160 (mmHg)	Yes	618 (40,1%)	146	0.98 (0.79; 1.20)	1.02 (0.83; 1.26)
	No	925 (59,9%)	226		
Treated with ACE-I or ARB in patients with ACR ≥2.5 (males) or ≥3.5 (females) (mg/mmol)	Yes	762 (64,2%)	182	**0.70 (0.56; 0.88)**	**0.75 (0.59; 0.94)**
	No	425 (35,8%)	130		
Treatment intensification in patients with ACR ≥2.5 (males) or ≥3.5 (females) (mg/mmol)	Yes	143 (15,1%)	31	0.77 (0.53; 1.13)	0.79 (0.54; 1.15)
	No	806 (84,9%)	217		

Bold text indicates significant hazards ratio (cox regression); * - adjusted for baseline morbidity; ** - adjusted for baseline morbidity and comorbidity, baseline related risk factor level, baseline medications and individual patients characteristics (age, gender, duration of diabetes).

### Quality indicators measuring current treatment status

**Figure 1:**
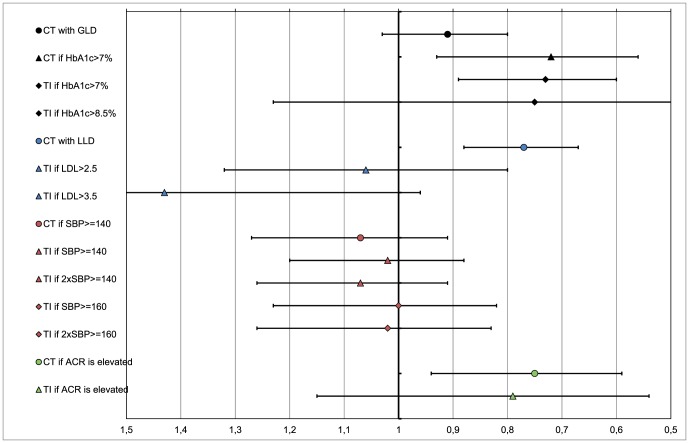
Predictive value of quality indicators on a composite of cardiovascular events and all-cause death. Legend: The predictive value is represented as the hazard of event occurrence in patients treated as defined by the quality indicator in comparison to those not treated as such after adjusting on patients characteristics, that is age, gender, duration of diabetes, baseline risk factor level, baseline treatment status (glucose-, blood pressure-, lipid-lowering drugs), history of malignancies, and history of psychological disorders. CT – current treatment; TI – treatment intensification; GLD – glucose-lowering drugs; LLD – lipid-lowering drugs.

Being treated with lipid and albuminuria lowering drugs was significantly associated with a lower risk of hard outcomes (Figure 1). Being treated with glucose lowering drugs was significantly associated with a lower risk of hard outcomes only in patients with an elevated HbA1c level. Being treated with blood pressure lowering drugs was not significantly associated with hard outcomes.

### Quality indicators measuring treatment intensification when indicated

Treatment intensification with glucose lowering drugs was significantly associated with a lower risk of hard outcomes (Figure 1). In turn, treatment intensification with lipid-, blood pressure- and albuminuria-lowering drugs was not significantly associated with a risk of hard outcomes.

## Discussion

This study shows that the quality indicators measuring current treatment status with lipid- and albuminuria-lowering drugs predicted a lower risk of hard cardiovascular outcomes in patients with diabetes in general practice. For the indicators measuring treatment intensification, only the one focusing on glucose lowering treatment intensification predicted a lower risk of hard outcomes. None of the quality indicators measuring blood pressure lowering treatment or treatment intensification were predictive of hard outcomes.

Quality indicators are increasingly used for measuring the quality of diabetes care to improve providers performance and patients health [23], e.g., in the Quality and Outcome Framework in the United Kingdom [7]. To our knowledge, this is the first longitudinal study assessing which treatment quality indicators for patients with diabetes are predictive of hard outcomes in primary care. Especially when indicators are used by policy makers for public reporting or by insurance companies for rewarding providers, it is essential to identify quality indicators that directly reflect providers' actions and lead to benefits in patient outcomes. Due to bias in the indicator definition or inadequate assessment of treatment quality not all of the quality indicators predict better patient outcomes in practice. Alternatively, the evidence for some treatments being beneficial may not be that straightforward when translated to actual practice, where patients are often older and have more comorbidity in comparison to the trial populations. Whatever the reason, this study shows that such treatment quality indicators should not be used as they are defined and measured nowadays.

It is assumed that both treatment and treatment intensification when indicated will lead to better intermediate outcomes and to a lower risk of cardiovascular outcomes [16],[17]. Our composite outcome included a range of macrovascular and microvascular complications and all-cause death, which were proven to benefit from adequate risk factor treatment [1]–[4]. The indicators measuring current treatment status are relatively easy to calculate using routinely collected data from clinical practice. They are included in several national indicator sets for quality assessment [7]–[9]. Previously, the lipid lowering treatment indicator showed an association with intermediate outcomes in patients with diabetes [6]. Our study adds to this knowledge by showing that this indicator also predicts a lower risk of hard outcomes. Apparently, assessing whether patients with diabetes are being treated with lipid lowering drugs at one point in time is a good measure of adequate treatment. Furthermore, the glucose lowering treatment indicator was previously found to be only predictive of better intermediate outcomes in a restricted population of patients with an elevated HbA1c level [5]. In the current study, we found the same need for restriction when looking at hard outcomes. Since there is no need to prescribe glucose lowering treatment to patients who are well-controlled on diet, the eligible population for this treatment indicator should be restricted to uncontrolled patients. An interesting finding of our study was that the indicator measuring albuminuria lowering treatment status was predictive of a lower risk of hard outcomes, where it previously showed no association with albuminuria control [6]. This finding may reflect the fact that antihypertensive treatment with drugs acting on renin-angiotensin-system is advised in patients with increased albuminuria. These drugs appear to have a specific cardiovascular protection beyond their effect on one single risk factor [4]. The quality indicator of blood pressure lowering treatment status was not predictive of hard outcomes. Previously, it was found that such an indicator was also not associated with blood pressure control [6]. These findings may seem surprising since clinical trials showed beneficial effect of treatment on blood pressure and cardiovascular outcomes [3],[26]. Quality indicators measuring blood pressure treatment, as they are defined, may be too simplistic. They do not account for any heterogeneity in the patient population or indication for treatment, and disregard intrapersonal blood pressure variability. This could be partly solved by making more specific indicators (e.g. for specific age groups). In addition, the lack of association with hard outcomes may be the result of including patients without further treatment intensification when their blood pressure deteriorates. It has been shown in a simulation study that patients with diabetes and hypertension may need many intensifications to keep their blood pressure level under control [27].

The alternative indicators for treatment quality in our study measured whether patients with diabetes and an elevated risk factor level received treatment intensification. We found that only the indicator measuring glucose lowering treatment intensification showed predictive value on a lower risk of hard outcomes. Previously, it was shown that this indicator was predictive of better intermediate outcome of glycemic control [5],[12]. Moreover, the indicator measuring treatment intensification with lipid lowering drugs previously also showed a predictive association with better cholesterol control. It was somewhat unexpected that only the intensification indicator for glucose lowering treatment was predictive of a lower risk of hard outcomes, since glycemic control appears to have less impact on cardiovascular outcomes comparing with blood pressure and cholesterol control [1],[16]. One could argue that unmeasured confounding may explain the association between glucose lowering treatment intensification and hard outcomes. That is, that sicker patients with more comorbidity, who will have poorer outcomes, may be less aggressively treated for their diabetes. However, in a previous study we found no evidence that comorbid conditions decrease the likelihood to intensify medication treatment in patients with diabetes [28]. Moreover, the absence of associations between the indicators measuring lipid- and blood pressure-lowering treatment intensification and hard outcomes also makes this explanation less likely. An alternative explanation for the difference in associations for these indicators may be that they do not adequately reflect fluctuations in the quality of drug treatment over time. In the long run, patients may deteriorate if further intensification is not prescribed when needed. Health care providers' behavior is not necessarily consistent regarding treatment intensification over time. Previously, it was shown that providers are more prone to intensify glucose lowering than blood pressure- or cholesterol-lowering treatment [29]. For blood pressure lowering treatment an alternative indicator has been suggested, which assesses the number of treatment intensifications longitudinally in relation to the number of occasions where the blood pressure level was elevated [30]. This indicator showed good prediction of intermediate outcomes [31], but has not yet been tested against hard outcomes.

Finally, non-adherence to treatment may also explain a lack of association between quality indicators measuring treatment intensification and hard outcomes. Non-adherence is common among patients with diabetes and associated with a higher risk of cardiovascular outcomes [32]. When clinicians are not aware of the non-adherence, they are likely to intensify treatment in such patients. This phenomenon has been observed for lipid- and blood-pressure lowering treatment but not for glucose lowering treatment [32]–[34]. On the other hand, a small observational study using alternative indicator measuring treatment intensification showed an improvement in blood pressure control regardless of the patient's adherence level [35].

### Strengths and limitations

This study was conducted in a large cohort of patients with diabetes from a primary care setting in the north of the Netherlands. We lost 8% of patients who could not be linked to the hospital data because they changed their place of residence. The population of our cohort consists mainly of individuals of West-European origin, which may influence a risk of vascular events occurrence [36]. Observational studies are susceptible to a number of biases. For quality indicators, it is assumed that all eligible patients have an indication for treatment or treatment intensification. However, patients who are more likely to get the outcome may also be more likely to get treatment, which could lead to unexpected associations between the indicator and the outcome. Therefore, we adjusted the tested associations for baseline treatment status, morbidity and other patients characteristics which may be possible confounders. Our adjustment on baseline morbidity, however, was based on morbidity history data from primary care records, which might not be complete [37]. We tried to minimize incompleteness by enriching the diagnoses data by manually coding text descriptions. Although we adjusted the tested association for possible confounders, there may be unmeasured confounding, partly related to patient behavior. We did not adjust the tested associations for non-adherence or lifestyle, which may lead to underestimation of the associations between indicators and outcomes. Another limitation of our study is that the data on cardiovascular outcomes in 2010 was incomplete, because one regional hospital did not provide data to the Dutch Hospital Data register that year.

## Conclusions

This study demonstrated that treatment quality indicators are not always reliable instruments for measuring diabetes treatment quality as observed in primary care. The quality indicators measuring lipid- and albuminuria-lowering treatment status can be considered for implementation into quality indicator sets, since these indicators appear to result in less cardiovascular outcomes. The indicator measuring glucose lowering treatment status should be restricted to include only patients with an elevated HbA1c. The indicators measuring blood pressure lowering treatment status cannot be used as such, since they are not related to cardiovascular outcomes. To measure quality of blood pressure lowering treatment, the use of indicators assessing treatment over time needs further exploration. Finally, indicators measuring treatment intensification at one point of time may be helpful for quality improvement initiatives, to show where action is needed, but they do not reliably predict treatment quality over time. Since treatment over time is not only associated with patients' well-being and disease burden, but also with health economics, repeated measurement of treatment quality is needed for chronic diseases.

## Supporting Information

Table S1 **The included drug classes for measuring treatment intensification.**
(DOCX)

Table S2 **The list of events included for the composite outcome.**
(DOCX)

Table S3 **Baseline cardiovascular morbidity and comorbidity.**
(DOCX)

Table S4 **Patients characteristics per quality indicator.**
(DOCX)
