# Supplementary material for: Do Treatment Quality Indicators Predict Cardiovascular Outcomes in Patients with Diabetes?
Source: PLoS One. 2013 Oct 30;8(10):e78821. doi: 10.1371/journal.pone.0078821 (PMC3813585; doi:10.1371/journal.pone.0078821)
Supplement: Table S1 — The included drug classes for measuring treatment intensification. (DOCX) [file pone.0078821.s001.docx]

**Table S1.** Included drug classes for measuring treatment intensification

| **Type of drug treatment** | **Included drug classes** |
| --- | --- |
| glucose-lowering treatment | biguanides |
|  | sulfonylureas |
|  | α-glucosidase inhibitors |
|  | thiazolidinediones |
|  | dipeptidyl-peptidase-4-inhibitors |
|  | insulins |
|  | other blood glucose–lowering drugs (e.g. nateglinide, exenatide) |
| lipid lowering treatment | statins |
|  | fibrates |
|  | bile acid sequestrants |
|  | nicotinic acid and derivatives |
|  | other lipid modifying drugs (omacor, ezitimib, ezetrol) |
| blood pressure lowering treatment | centrally-acting antihypertensives |
|  | diuretics |
|  | beta blockers |
|  | calcium channel blockers |
|  | drugs acting on the renin-angiotensin system |
| albuminuria lowering treatment | drugs acting on the renin-angiotensin system |
