# Supplementary material for: Do Treatment Quality Indicators Predict Cardiovascular Outcomes in Patients with Diabetes?
Source: PLoS One. 2013 Oct 30;8(10):e78821. doi: 10.1371/journal.pone.0078821 (PMC3813585; doi:10.1371/journal.pone.0078821)
Supplement: Table S2 — The list of events included for the composite outcome. (DOCX) [file pone.0078821.s002.docx]

**Table S2.** List of events included for the composite outcome

|  | **ICPC-1** | **ICD-9-CM diagnose codes** | **ICD-9-CM procedure codes** | **Classification of the specialized medical procedures** |
| --- | --- | --- | --- | --- |
| **Cardiovascular outcomes** |  |  |  |  |
| Myocardial Infarction | K75 | 410 |  |  |
| Ischemic Heart Disease | K76 | 411 |  |  |
| Angina Pectoris | K74 | 413 |  |  |
| Other forms of chronic ischemic heart disease | K76 | 414 |  |  |
| Percutaneous transluminal coronary angioplasty | GT |  | 00.66 | 8-8370, 8-8374 |
| Endarterectomy of coronary vessels | - |  | - | 5-360 |
| Coronary artery bypass surgery | GT |  | 36.1 | 5-361 |
| Other shunt or vascular bypass of coronary arteries | - |  |  | 5-362, 5-363 |
| **Cerebrovascular outcomes** |  |  |  |  |
| Subarachnoid hemorrhage | K90 | 430 |  |  |
| Intracerebral hemorrhage | K90 | 431 |  |  |
| Other and unspecified intracranial hemorrhage | K90 | 432 |  |  |
| Occlusion and stenosis of precerebral arteries | - | 433 |  |  |
| Occlusion of cerebral arteries | K90 | 434 |  |  |
| Transient cerebral ischemia | K89 | 435 |  |  |
| Acute, but ill-defined, cerebrovascular disease | K91 | 436 |  |  |
| Other and ill-defined cerebrovascular disease | K91 | 437 |  |  |
| Late effects of cerebrovascular disease | K91 | 438 |  |  |
| Diabetic retinopathy | F83 | 362(0-2) |  |  |
| Percutaneous transluminal cerebrovascular angioplasty | GT |  | 0.61, 0.62, 0.63, 0.64, 0.65 | 8-83601, 8-8362(1,2), 8-83651 |
| Endarterectomy of (pre)cerebral arteries | - |  | 38.11, 38.12 | 5-381(0,1) |
| Other shunt or vascular (pre)cerebral arteries | - |  | 39.22 | 5-3920 |
| **Peripheral vascular outcomes** |  |  |  |  |
| Diabetes nephropathy | GT | 250.4 |  |  |
| Diabetes with neurological manifestations | N94.2 | 250.6 |  |  |
| Amputation of lower limb | GT |  | 84.1 | 5-84(5-8) |
| Other shunt or vascular bypass | GT |  | 39.2 | 5-392(1-9) |
| Angioplasty or atherectomy of other non-coronary vessel(s) | GT |  | 39.50 | 8-8360(0,2-9), 8-8362(0,3-9), 8-836(3,4), 8-8365(0,2-9), 8-836(7-9), 8-837(1-3,5-9) |
| Chronic kidney disease | U99.1 | 585 |  |  |
| Dialysis | GT |  | 39.95, 54.98 | 8-853, 8-860 |
| Kidney transplantation | GT |  | 55.6 | 5-555 |

GT = GIANTT specific code
