# Supplementary material for: Do Treatment Quality Indicators Predict Cardiovascular Outcomes in Patients with Diabetes?
Source: PLoS One. 2013 Oct 30;8(10):e78821. doi: 10.1371/journal.pone.0078821 (PMC3813585; doi:10.1371/journal.pone.0078821)
Supplement: Table S3 — Baseline cardiovascular morbidity and comorbidity. (DOCX) [file pone.0078821.s003.docx]

**Table S3.** Baseline cardiovascular morbidity and comorbidity

|  | **ICPC-1 code (or GIANTT code)** |
| --- | --- |
| **Cardiovascular morbidity** |  |
| Angina pectoris | K74 |
| Myocardial infarction | K75,76 |
| Heart failure | K77 |
| Left ventricular hypertrophy | GT |
| Coronary artery bypass grafting | GT |
| Percutaneous transluminal coronary angioplasty | GT |
| Stroke | K90 |
| Cerebral ischemia | K89 |
| Retinopathy | F83 |
| Peripheral vascular disease | K92 |
| Atherosclerosis | K91 |
| Percutaneous transluminal angioplasty | GT |
| Peripheral bypass | GT |
| Aneurism aorta | K99.1 |
| Renal failure | U99.1 |
| Diabetes nephropathy | GT |
| Terminal dialysis | GT |
| Kidney transplantation | GT |
| Diabetes neuropathy | N94.2 |
| Diabetic foot and related amputations | GT |
| **Malignancies** |  |
| Metastases | A79 |
| Malignancies related to blood and blood forming organs | B72, B72.1, B72.2, B73, B74 |
| Malignancies related to gastrointestinal tract | D74, D75, D76, D77, D77.177.2, 77.3, D77.4 |
| Malignancy related to eye | F74.1 |
| Malignancy related to ear | H75.1 |
| Malignancy related to cardiovascular system | K72.1 |
| Malignancy related to musculoskeletal system | L71.1 |
| Malignancy related to neurological system | N74 |
| Malignancy related to respiratory system | R84, R85 |
| Malignancy related to skin | S77, S77.1, S77.2, S77.3, S77.4 |
| Malignancy related to thyroid | T71 |
| Malignancy related to urinary system | U75, U76, U77 |
| Malignancy related to female genital system (including breast) | X75, X76, X76.1, X77, X77.1, X77.2 |
| Malignancy related to male genital system | Y77, Y78, Y78.1, Y78.2, Y78.3 |
| **Psychological disorders** |  |
| Any psychological disorder | P70 - P99 |

GT = GIANTT specific code
