# Supplementary material for: Do Treatment Quality Indicators Predict Cardiovascular Outcomes in Patients with Diabetes?
Source: PLoS One. 2013 Oct 30;8(10):e78821. doi: 10.1371/journal.pone.0078821 (PMC3813585; doi:10.1371/journal.pone.0078821)
Supplement: Table S4 — Patients characteristics per quality indicator. (DOCX) [file pone.0078821.s004.docx]

**Table S4.** Patients characteristics per quality indicator

| **Patient characteristics** | **Number of patients eligible for quality assessment** | **Mean ± SD / Number of patients who are treated according to QI** | **Mean ± SD / Number of patients who are not treated according to QI** | **Standardized difference*** |
| --- | --- | --- | --- | --- |
| **Treated with glucose lowering drugs** | 8455 | 6754 | 1701 |  |
| Male gender | 4007 | 3197 | 810 | 1,87 |
| Age (years) | 8455 | 66.9 ± 11.8 | 66.7 ± 12.6 | 0,02 |
| Duration of diabetes (years) | 8455 | 6754 | 1701 | 1,88 |
| < 3 | 2948 | 2091 | 857 | 1,10 |
| 3-10 | 4292 | 3603 | 689 | 2,40 |
| > 10 | 1215 | 1060 | 155 | 2,95 |
| Baseline HbA1c level (%) | 8455 | 7.0 ± 1.0 | 6.4 ± 0.9 | 0,63 |
| Treated with lipid lowering drugs | 6510 | 5439 | 1071 | 2,34 |
| Treated with blood pressure lowering drugs | 6502 | 5313 | 1189 | 2,10 |
| History of cardiovascular morbidity | 1778 | 1444 | 334 | 2,04 |
| History of malignancy | 607 | 471 | 136 | 1,65 |
| History of psychological comorbidity | 327 | 239 | 88 | 1,26 |
| **Treated with glucose lowering drugs in patients with HbA1c > 7 (%)** | 2700 | 2462 | 238 |  |
| Male gender | 1296 | 1179 | 117 | 3,86 |
| Age (years) | 2700 | 66.7 ± 12.3 | 67.1 ± 13.6 | 0,03 |
| Duration of diabetes (years) | 2700 | 2462 | 238 | 3,92 |
| < 3 | 652 | 587 | 65 | 3,59 |
| 3-10 | 1475 | 1350 | 125 | 4,03 |
| > 10 | 573 | 525 | 48 | 4,07 |
| Baseline HbA1c level (%) | 2700 | 7.9 ± 1.0 | 8.0 ± 1.1 | -0,10 |
| Treated with lipid lowering drugs | 2118 | 1977 | 141 | 4,75 |
| Treated with blood pressure lowering drugs | 2081 | 1920 | 161 | 4,30 |
| History of cardiovascular morbidity | 559 | 506 | 53 | 3,72 |
| History of malignancy | 175 | 154 | 21 | 3,10 |
| History of psychological comorbidity | 112 | 99 | 13 | 3,19 |

| **Patient characteristics** | **Number of patients eligible for quality assessment** | **Mean ± SD / Number of patients who are treated according to QI** | **Mean ± SD / Number of patients who are not treated according to QI** | **Standardized difference*** |
| --- | --- | --- | --- | --- |
| **Treatment intensification in patients with HbA1c > 7 (%)** | 2455 | 848 | 1607 |  |
| Male gender | 1206 | 444 | 762 | -0,47 |
| Age (years) | 2455 | 64.9 ± 12.0 | 67.6 ± 12.6 | -0,22 |
| Duration of diabetes (years) | 2455 | 1607 | 848 | 0,74 |
| < 3 | 637 | 310 | 327 | -0,05 |
| 3-10 | 1345 | 452 | 893 | -0,57 |
| > 10 | 473 | 86 | 387 | -0,99 |
| Baseline HbA1c level (%) | 2455 | 8.0 ± 1.1 | 7.9 ± 0.9 | 0,10 |
| Treated with lipid lowering drugs | 1920 | 696 | 1224 | -0,49 |
| Treated with blood pressure lowering drugs | 1871 | 621 | 1250 | -0,58 |
| History of cardiovascular morbidity | 455 | 138 | 317 | -0,67 |
| History of malignancy | 156 | 54 | 102 | -0,54 |
| History of psychological comorbidity | 102 | 37 | 65 | -0,49 |
| **Treatment intensification in patients with HbA1c > 8.5 (%)** | 401 | 145 | 256 |  |
| Male gender | 211 | 81 | 130 | -0,42 |
| Age (years) | 401 | 62.3 ± 13.5 | 63.9 ± 13.8 | -0,12 |
| Duration of diabetes (years) | 401 | 145 | 256 | -0,49 |
| < 3 | 94 | 59 | 35 | 0,59 |
| 3-10 | 205 | 61 | 144 | -0,68 |
| > 10 | 102 | 25 | 77 | -0,83 |
| Baseline HbA1c level (%) | 401 | 9.9 ± 1.4 | 9.6 ± 1.1 | 0,24 |
| Treated with lipid lowering drugs | 299 | 116 | 183 | -0,41 |
| Treated with blood pressure lowering drugs | 279 | 98 | 181 | -0,52 |
| History of cardiovascular morbidity | 86 | 30 | 56 | -0,53 |
| History of malignancy | - | - | - | - |
| History of psychological comorbidity | - | - | - | - |

| **Patient characteristics** | **Number of patients eligible for quality assessment** | **Mean ± SD / Number of patients who are treated according to QI** | **Mean ± SD / Number of patients who are not treated according to QI** | **Standardized difference*** |
| --- | --- | --- | --- | --- |
| **Treated with lipid lowering drugs** | 6471 | 4360 | 2111 |  |
| Male gender | 3080 | 2119 | 961 | 0,95 |
| Age (years) | 6471 | 65.8 ± 10.8 | 68.2 ± 13.1 | -0,20 |
| Duration of diabetes (years) | 6471 | 4360 | 2111 | 0,86 |
| < 3 | 2310 | 1556 | 754 | 0,86 |
| 3-10 | 3276 | 2231 | 1045 | 0,91 |
| > 10 | 885 | 573 | 312 | 0,70 |
| Baseline LDL-C level (mmol/l) | 6471 | 2.2 ± 0.9 | 2.8 ± 0.9 | -0,67 |
| Treated with glucose lowering drugs | 5553 | 3884 | 1669 | 1,03 |
| Treated with blood pressure lowering drugs | 4968 | 3487 | 1481 | 1,05 |
| History of cardiovascular morbidity | 1358 | 992 | 366 | 1,26 |
| History of malignancy | 462 | 294 | 168 | 0,64 |
| History of psychological comorbidity | 259 | 171 | 88 | 0,78 |
| **Treatment intensification in patients with**  **LDL-C > 2.5 (mmol/l)** | 2239 | 375 | 1864 |  |
| Male gender | 982 | 179 | 803 | -0,99 |
| Age (years) | 2239 | 63.3 ± 11.6 | 67.1 ± 12.4 | -0,32 |
| Duration of diabetes (years) | 2239 | 375 | 1864 | -1,03 |
| < 3 | 864 | 162 | 702 | -0,98 |
| 3-10 | 1079 | 174 | 905 | -1,05 |
| > 10 | 296 | 39 | 257 | -1,12 |
| Baseline LDL-C level (mmol/l) | 2239 | 3.7 ± 0.7 | 3.3 ± 0.6 | 0,61 |
| Treated with glucose lowering drugs | 1769 | 303 | 1466 | -1,02 |
| Treated with blood pressure lowering drugs | 1619 | 258 | 1361 | -1,05 |
| History of cardiovascular morbidity | 378 | 62 | 316 | -1,04 |
| History of malignancy | 160 | 20 | 140 | -1,13 |
| History of psychological comorbidity | 91 | 18 | 73 | -0,95 |

| **Patient characteristics** | **Number of patients eligible for quality assessment** | **Mean ± SD / Number of patients who are treated according to QI** | **Mean ± SD / Number of patients who are not treated according to QI** | **Standardized difference*** |
| --- | --- | --- | --- | --- |
| **Treatment intensification in patients with**  **LDL-C > 3.5 (mmol/l)** | 683 | 184 | 499 |  |
| Male gender | 285 | 85 | 200 | -0,68 |
| Age (years) | 683 | 64.2 ± 12.6 | 68.0 ± 12.4 | -0,30 |
| Duration of diabetes (years) | 683 | 85 | 200 | -0,39 |
| < 3 | 283 | 84 | 199 | -0,69 |
| 3-10 | 326 | 86 | 240 | -0,78 |
| > 10 | 74 | 14 | 60 | -0,98 |
| Baseline LDL-C level (mmol/l) | 683 | 4.2 ± 0.6 | 4.1 ± 0.5 | 0,18 |
| Treated with glucose lowering drugs | 501 | 144 | 357 | -0,71 |
| Treated with blood pressure lowering drugs | 483 | 123 | 360 | -0,80 |
| History of cardiovascular morbidity | 105 | 29 | 76 | -0,74 |
| History of malignancy | 57 | 11 | 46 | -0,97 |
| History of psychological comorbidity | 37 | 11 | 26 | -0,68 |
| **Treated with blood pressure lowering drugs in patients with SBP ≥140 (mmHg)** | 4937 | 3915 | 1022 |  |
| Male gender | 2222 | 1722 | 500 | 1,64 |
| Age (years) | 4937 | 69.5 ± 10.9 | 66.9 ± 12.3 | 0,22 |
| Duration of diabetes (years) | 4937 | 3915 | 1022 | 1,82 |
| < 3 | 1638 | 1264 | 374 | 1,61 |
| 3-10 | 2536 | 2033 | 503 | 1,92 |
| > 10 | 763 | 618 | 145 | 2,01 |
| Baseline SBP level (mmHg) | 4937 | 157.5 ± 16.0 | 152.4 ± 13.5 | 0,34 |
| Treated with glucose lowering drugs | 4225 | 3399 | 826 | 1,95 |
| Treated with lipid lowering drugs | 3750 | 3082 | 668 | 2,16 |
| History of cardiovascular morbidity | 1048 | 916 | 132 | 2,98 |
| History of malignancy | 380 | 307 | 73 | 1,99 |
| History of psychological comorbidity | 166 | 124 | 42 | 1,39 |

| **Patient characteristics** | **Number of patients eligible for quality assessment** | **Mean ± SD / Number of patients who are treated according to QI** | **Mean ± SD / Number of patients who are not treated according to QI** | **Standardized difference*** |
| --- | --- | --- | --- | --- |
| **Treatment intensification in patients with SBP ≥140 (mmHg)** | 4864 | 1004 | 3860 |  |
| Male gender | 2184 | 453 | 1731 | -0,93 |
| Age (years) | 4864 | 68.6 ± 11.2 | 69.1 ± 11.3 | -0,04 |
| Duration of diabetes (years) | 4864 | 1004 | 3860 | -0,93 |
| < 3 | 1620 | 351 | 1269 | -0,91 |
| 3-10 | 2496 | 504 | 1992 | -0,94 |
| > 10 | 748 | 149 | 599 | -0,95 |
| Baseline SBP level (mmHg) | 4864 | 164.0 ± 17.9 | 154.4 ± 14.3 | 0,59 |
| Treated with glucose lowering drugs | 3693 | 780 | 2913 | -0,92 |
| Treated with lipid lowering drugs | 4161 | 859 | 3302 | -0,93 |
| History of cardiovascular morbidity | 973 | 210 | 763 | -0,91 |
| History of malignancy | 379 | 75 | 304 | -0,95 |
| History of psychological comorbidity | 164 | 35 | 129 | -0,91 |
| **Treatment intensification in patients with 2 sequential SBP tests ≥140 (mmHg)** | 4146 | 982 | 3164 |  |
| Male gender | 1838 | 441 | 1397 | -0,84 |
| Age (years) | 4146 | 68.7 ± 11.0 | 69.5 ± 10.9 | -0,07 |
| Duration of diabetes (years) | 4146 | 982 | 3164 | -0,85 |
| < 3 | 1376 | 359 | 1017 | -0,79 |
| 3-10 | 2112 | 479 | 1633 | -0,88 |
| > 10 | 661 | 145 | 516 | -0,90 |
| Baseline SBP level (mmHg) | 4146 | 69.5 ± 10.9 | 67.0 ± 12.4 | 0,21 |
| Treated with glucose lowering drugs | 3604 | 838 | 2766 | -0,86 |
| Treated with lipid lowering drugs | 3205 | 774 | 2431 | -0,84 |
| History of cardiovascular morbidity | 835 | 212 | 623 | -0,81 |
| History of malignancy | 320 | 78 | 242 | -0,83 |
| History of psychological comorbidity | 138 | 32 | 106 | -0,87 |

| **Patient characteristics** | **Number of patients eligible for quality assessment** | **Mean ± SD / Number of patients who are treated according to QI** | **Mean ± SD / Number of patients who are not treated according to QI** | **Standardized difference*** |
| --- | --- | --- | --- | --- |
| **Treatment intensification in patients with**  **SBP ≥160 (mmHg)** | 1947 | 598 | 1349 |  |
| Male gender | 779 | 242 | 537 | -0,65 |
| Age (years) | 1947 | 69.5 ± 11.1 | 70.8 ± 10.7 | -0,12 |
| Duration of diabetes (years) | 1947 | 598 | 1349 | -0,66 |
| < 3 | 628 | 205 | 423 | -0,60 |
| 3-10 | 992 | 296 | 696 | -0,68 |
| > 10 | 327 | 97 | 230 | -0,69 |
| Baseline SBP level (mmHg) | 1947 | 175.1 ± 14.7 | 170.1 ± 12.2 | 0,37 |
| Treated with glucose lowering drugs | 1658 | 514 | 1144 | -0,65 |
| Treated with lipid lowering drugs | 1459 | 474 | 985 | -0,60 |
| History of cardiovascular morbidity | 410 | 118 | 292 | -0,71 |
| History of malignancy | 160 | 39 | 121 | -0,83 |
| History of psychological comorbidity | - | - | - | - |
| **Treatment intensification in patients with 2 sequential SBP tests ≥160 (mmHg)** | 1543 | 618 | 925 |  |
| Male gender | 616 | 255 | 361 | -0,32 |
| Age (years) | 1543 | 70.0 ± 10.3 | 71.2 ± 10.3 | -0,12 |
| Duration of diabetes (years) | 1543 | 618 | 925 | -0,36 |
| < 3 | 481 | 218 | 263 | -0,18 |
| 3-10 | 807 | 297 | 510 | -0,47 |
| > 10 | 255 | 103 | 152 | -0,35 |
| Baseline SBP level (mmHg) | 1543 | 175.5 ± 14.7 | 171.8 ± 12.4 | 0,27 |
| Treated with glucose lowering drugs | 1361 | 545 | 816 | -0,36 |
| Treated with lipid lowering drugs | 1185 | 499 | 686 | -0,29 |
| History of cardiovascular morbidity | 334 | 139 | 195 | -0,31 |
| History of malignancy | 116 | 42 | 74 | -0,49 |
| History of psychological comorbidity | - | - | - | - |

| **Patient characteristics** | **Number of patients eligible for quality assessment** | **Mean ± SD / Number of patients who are treated according to QI** | **Mean ± SD / Number of patients who are not treated according to QI** | **Standardized difference*** |
| --- | --- | --- | --- | --- |
| **Treated with ACE-I or ARB in patients with ACR ≥2.5 (males) or ≥3.5 (females) (mg/mmol)** | 1187 | 762 | 425 |  |
| Male gender | 656 | 437 | 219 | 0,81 |
| Age (years) | 1187 | 69.2 ± 11.1 | 70.5 ± 11.6 | -0,11 |
| Duration of diabetes (years) | 1187 | 762 | 425 | 0,67 |
| < 3 | 343 | 222 | 121 | 0,70 |
| 3-10 | 634 | 398 | 236 | 0,59 |
| > 10 | 210 | 142 | 68 | 0,88 |
| Baseline ACR level (mg/mmol) | 1187 | 17.7 ± 30.4 | 12.6 ± 17.4 | 0,21 |
| Treated with glucose lowering drugs | 1069 | 694 | 375 | 0,71 |
| Treated with lipid lowering drugs | 914 | 624 | 290 | 0,92 |
| History of cardiovascular morbidity | 346 | 239 | 107 | 0,97 |
| History of malignancy | 110 | 64 | 46 | 0,36 |
| History of psychological comorbidity | - | - | - | - |
| **Treatment intensification in patients with ACR ≥ 2.5 (males) or ≥ 3.5 (females) (mg/mmol)** | 949 | 143 | 806 |  |
| Male gender | 523 | 85 | 438 | -1,04 |
| Age (years) | 949 | 67.0 ± 12.3 | 69.9 ± 11.4 | -0,24 |
| Duration of diabetes (years) | 949 | 143 | 806 | -1,07 |
| < 3 | 282 | 45 | 237 | -1,05 |
| 3-10 | 514 | 75 | 439 | -1,08 |
| > 10 | 153 | 23 | 130 | -1,07 |
| Baseline ACR level (mg/mmol) | 949 | 20.4 ± 34.1 | 13.3 ± 19.1 | 0,26 |
| Treated with glucose lowering drugs | 848 | 130 | 718 | -1,07 |
| Treated with lipid lowering drugs | 720 | 116 | 604 | -1,05 |
| History of cardiovascular morbidity | 258 | 44 | 214 | -1,02 |
| History of malignancy | 91 | 12 | 79 | -1,12 |
| History of psychological comorbidity | - | - | - | - |

* - standardized difference compares the difference in means in units of the pooled standard deviation. A larger value of the standardized difference designates more inequality in baseline characteristics of patients receiving treatment according to a quality indicator compared to those not receiving the recommended treatment.
